# Principal Component Analysis (PCA) of Molecular Descriptors for Improving Permeation through the Blood–Brain Barrier of Quercetin Analogues

**DOI:** 10.3390/ijms25010192

**Published:** 2023-12-22

**Authors:** Nebojša Pavlović, Nastasija Milošević Sopta, Darko Mitrović, Dragana Zaklan, Ana Tomas Petrović, Nebojša Stilinović, Saša Vukmirović

**Affiliations:** 1 Department of Pharmacy, Faculty of Medicine, University of Novi Sad, Hajduk Veljkova 3, 21000 Novi Sad, Serbia; 904004d22@mf.uns.ac.rs (D.M.); dragana.zaklan@mf.uns.ac.rs (D.Z.); 2 Accelsiors CRO, Háros Street 103, 1222 Budapest, Hungary; nastasijamilosevic@gmail.com; 3 Department of Pharmacology, Toxicology and Clinical Pharmacology, Faculty of Medicine, University of Novi Sad, Hajduk Veljkova 3, 21000 Novi Sad, Serbia; ana.tomas@mf.uns.ac.rs (A.T.P.); nebojsa.stilinovic@mf.uns.ac.rs (N.S.); sasa.vukmirovic@mf.uns.ac.rs (S.V.)

**Keywords:** quercetin, neuroprotection, IPMK, molecular docking, blood–brain barrier, PCA

## Abstract

Despite its beneficial pharmacological effects in the brain, partly by modulating inositol phosphate multikinase (IPMK) activity, the therapeutic use of quercetin is limited due to its poor solubility, low oral bioavailability, and low permeability through the blood–brain barrier (BBB). We aimed to identify quercetin analogues with improved BBB permeability and preserved binding affinities towards IPMK and to identify the molecular characteristics required for them to permeate the BBB. Binding affinities of quercetin analogues towards IPMK were determined by molecular docking. Principal component analysis (PCA) was applied to identify the molecular descriptors contributing to efficient permeation through the BBB. Among 34 quercetin analogues, 19 compounds were found to form more stable complexes with IPMK, and the vast majority were found to be more lipophilic than quercetin. Using two distinct in silico techniques, insufficient BBB permeation was determined for all quercetin analogues. However, using the PCA method, the descriptors related to intrinsic solubility and lipophilicity (logP) were identified as mainly responsible for clustering four quercetin analogues (trihydroxyflavones) with the highest BBB permeability. The application of PCA revealed that quercetin analogues could be classified with respect to their structural characteristics, which may be utilized in further analogue syntheses and lead optimization of BBB-penetrating IPMK modulators as neuroprotective agents.

## 1. Introduction

Quercetin (3,3′,4′,5,7-pentahydroxyflavone) is a flavonoid ubiquitously present in vegetables and fruits, and it accounts for 60–75% of daily flavonoid intake [1]. Quercetin exists in the forms of aglycone and glycoside, which is hydrolyzed before absorption by lactase–phlorizin hydrolase. After absorption, it is bound to albumin and metabolized in the liver, with half-lives of quercetin itself and its metabolites ranging from 11 to 28 h [2]. It exerts a wide range of therapeutic effects, such as antioxidant, anti-inflammatory, antimicrobial, chemopreventive, cardioprotective, hypoglycemic, and neuroprotective [3]. There are numerous studies focused on the neuroprotective effects of quercetin, as many CNS disorders, such as Alzheimer’s disease (AD), Parkinson’s disease (PD), Huntington’s disease (HD), cognitive impairment, traumatic injury, and depression, are related to neurodegeneration caused by oxidative stress. Aggregation of amyloid β peptide (Aβ) and subsequent plaque formation are the hallmarks of AD, and they are associated with consecutive oxidative damage and neuroinflammation. It has been shown that quercetin protects neurons through oxidative stress reduction. In vitro and in vivo studies confirmed that quercetin also inhibits Aβ aggregation and tau phosphorylation, and it restores acetylcholine levels inhibiting the acetylcholinesterase [4]. Quercetin was also shown to attenuate neuroinflammation and to have positive effects on memory, learning, and other cognitive functions [5].

A recent structure–activity analysis confirmed that quercetin and some of its analogues can significantly affect the activity of inositol phosphate multikinase (IPMK) [6], which is very important in patients with HD, considering the very low levels of IPMK in striata of HD patients and HD animal models. Furthermore, a genome-wide association study of AD and immune-mediated disorders indicated a possible role of the IPMK gene in the etiology of neurodegeneration and neuroinflammation, suggesting the potential significance of IPMK in other brain disorders [7]. Therefore, further investigations on the activity of quercetin and structurally similar compounds related to IPMK modulation are of great importance.

Despite its beneficial effects, the therapeutic use of quercetin is limited due to its poor water solubility and low bioavailability after peroral use. In order to exert its effects in CNS, quercetin has to permeate the brain–blood barrier (BBB), which further limits its therapeutic potential. It has been shown that some quercetin metabolites have greater chances of passing through the BBB because of transporter-aided active transport. Hence, in the past several years, researchers have been developing different strategies to enhance quercetin bioavailability and brain distribution, from using novel pharmaceutical formulations to structural modifications of quercetin, including the formation of glucoside–sulfate conjugates, ionic complexes and the design and synthesis of its derivatives and structural analogues [8,9].

The use and relevance of in silico methods in drug discovery and development have intensively grown in the last decade, and they can be applied in drug discovery from target identification through hit identification to lead optimization and prediction of potential safety issues, thus increasing the success rate and reducing the costs [10,11]. Molecular descriptors have been widely used in computational drug discovery methods, representing the translation of certain chemical information of a molecular structure into a set of useful numeric values. Given a large number of molecular descriptors, the researchers often encounter problems such as elimination of irrelevant molecular descriptors, unfavorable descriptor:molecule ratio, and collinearity among descriptors. Principal component analysis (PCA), a multidimensional data analysis technique, has been thus developed to resolve these problems. It is commonly used in situations where data sets are large and complex and where the relationships between characteristics of a set of objects are not easily detected, as in QSAR studies. Therefore, the molecular descriptors are transformed to a new and reduced set of variables, called principal components (PCs), which are orthogonal to each other and arranged so that the first few carry the most useful information and preserve the variability of the original set as much as possible [12,13].

Accordingly, the aim of this study was to identify the analogues of quercetin with improved permeation through the blood–brain barrier and possessing high binding affinities towards IPMK that could be further investigated in depth as potential neuroprotective agents. Moreover, the molecular descriptors that contribute to better permeation through the BBB of quercetin analogues will be identified using the PCA strategy, which may be utilized in further drug design of potent IPMK modulators with neuroprotective effects.

## 2. Results

### 2.1. Molecular Docking of Quercetin and Its Analogues against IPMK

Molecular docking was performed to evaluate the binding affinities of quercetin analogues to the IPMK active site in comparison to the binding affinity of quercetin itself. Binding affinities towards IPMK for quercetin (compound **1**) and its analogues (compounds **2**–**35**) are presented in Table 1.

The interactions between IPMK protein and ligands were investigated through MolDock scores, which are expressed as kcal/mol. Binding energies of the tested quercetin analogues at the IPMK active site, i.e., the potential energies of the formed ligand–receptor complexes, were in the range from −91.827 kcal/mol for geraldol (compound **30**) to −72.415 kcal/mol for 3,5-dihydroxy-2-(4-phenyl)chromen-4-one (compound **25**). Quercetin had binding energy of −82.233 kcal/mol at the IPMK active site, and out of 34 quercetin analogues tested, 19 compounds had higher affinity towards the IPMK active site and built a more stable ligand–protein complex than quercetin.

**Figure 1:**
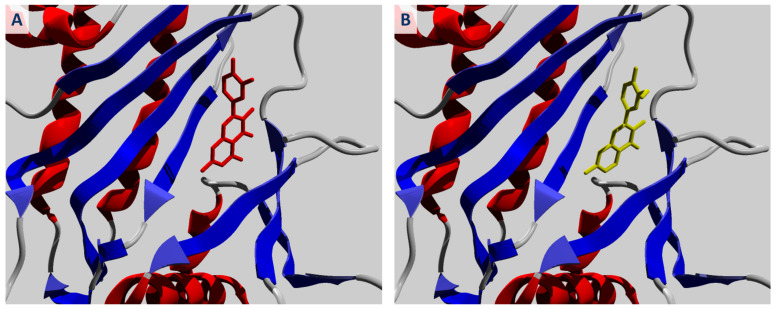
Binding mode of (**A**) quercetin (ZINC03869685) (red) and (**B**) geraldol (ZINC05732763) (yellow) in the active site of IPMK protein.

The molecular conformations of quercetin and geraldol, which had the strongest binding affinity for the IPMK active site, are shown in Figure 1. Both ligands have the similar conformations in the active site of IPMK protein, i.e., the same positions of the benzopyrone ring and phenyl group. However, the hydrophobic interactions between the methoxy group in the structure of geraldol and valine and leucine residues contributed to the stabilization of the ligand–protein complex.

### 2.2. In Silico Prediction of CNS Distribution

Molecular descriptors relevant to the membrane permeability were calculated for the tested compounds using the VolSurf+ mathematical models and they are presented in Table 1.

Within the range of molecular weights 270.24–360.32 g/mol, quercetin analogues had diverse polarities and abilities to permeate membranes. According to the calculated values of logP for quercetin analogues in n-octanol–water and cyclohexane–water systems, 27 and 29 compounds were found to be more lipophilic than quercetin, respectively. Accordingly, 28 quercetin analogues were predicted to possess lower aqueous solubility and 29 compounds to better permeate Caco-2 intestinal cells than quercetin (Table S1).

The calculated values of the logarithm of the blood–brain barrier distribution (LgBB) were lower than −0.5 for all quercetin analogues (range: −3.311 to −1.263), indicating poor brain permeation. Similarly to logP values, 27 analogues had higher values of LgBB than quercetin, with compound **33** (quercetin 3,4′-dimethyl ether) having the highest LgBB value.

**Figure 2:**
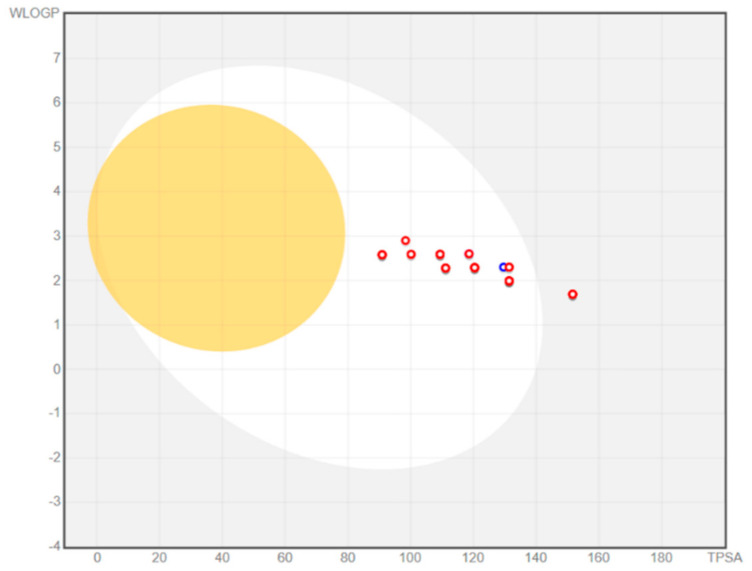
BOILED-Egg representation of the intestinal absorption and the permeation through blood–brain barrier for quercetin and its analogues.

Permeation of quercetin analogues through the BBB was also investigated using the “BOILED-Egg” method by SwissADME online tool, and the results are shown in Figure 2. BOILED-Egg is a graphical evaluation of the human intestinal absorption as a function of the position of the small molecule in the WLOGP vs. TPSA plot. Molecules located in the white region are predicted to be passively absorbed in the gastrointestinal tract, while the molecules in the “yolk” region should passively permeate the BBB. It can be observed that none of the analyzed compounds can pass through the BBB, but the majority of them can be passively absorbed in the intestines. Only four compounds (**2**, **3**, **6**, and **9**) were found to have low intestinal permeability, and all of these compounds had logP values lower than 2 (Table 1).

The potential of quercetin analogues to bind to P-glycoprotein (P-gp) was also investigated using the PgpRules server. Only two compounds, compound **33** (quercetin 3,4′-dimethyl ether) and compound **27** (3-O-methylquercetin), were shown to be substrates for P-gp, and none of the tested compounds showed P-gp-inhibitory activity (Table 1).

### 2.3. Principal Component Analysis (PCA)

Principal component analysis (PCA) was performed in order to gain an overview of the examined quercetin analogues for similarities and differences and to identify the outliers among the compounds with a 0.95 confidence level for T^2^ Hotelling limit. PCA resulted in a two-component model that explains more than 90% of the data variation.

**Figure 3:**
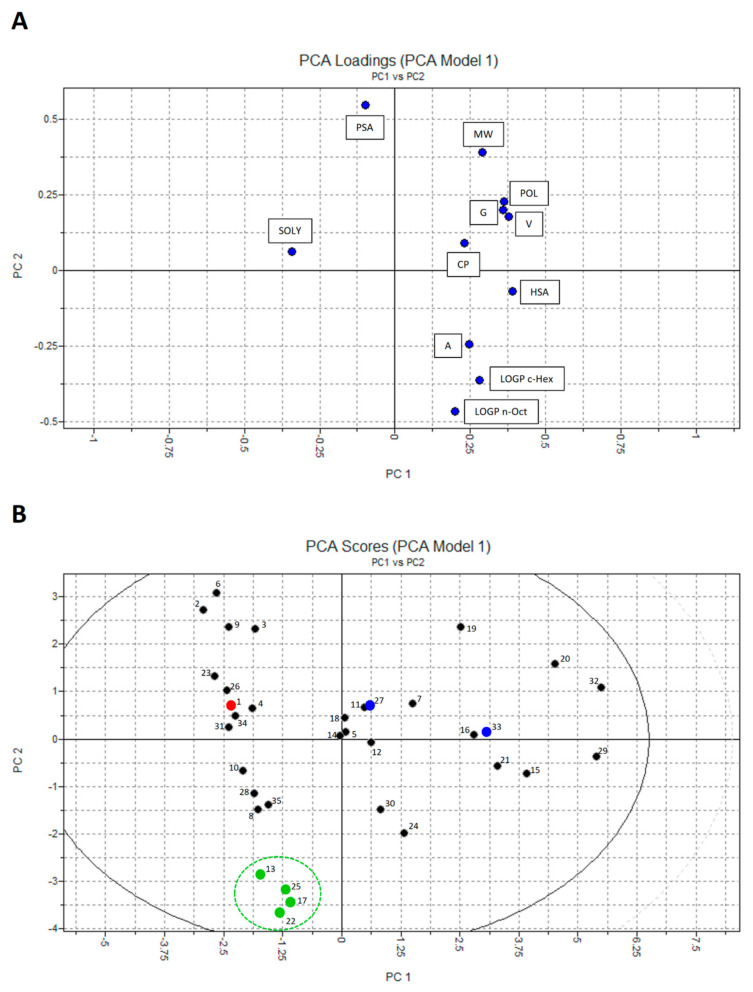
PCA loading plot (**A**) and score plot (**B**) of molecular descriptors for quercetin analogues. In the PCA score plot, quercetin (compound **1**) is marked as a red dot, compounds **13**, **17**, **22** and **25**, which all have higher brain permeation and are not P-gp substrates, are marked as green dots, and compounds **27** and **33**, which have higher brain permeation and are P-gp substrates, are marked as blue dots.

In Figure 3, the PCA score plot is presented (Figure 3B), i.e., the grouping pattern of the tested compounds, along with the PCA loading plot (Figure 3A), which indicates how much each descriptor contributes to the position of each compound in the score plot. The addition of more PCs did not change the distribution of the molecules in the score plot significantly. All tested compounds were inside the Hotelling T^2^ ellipse, indicating that there are no outliers among the tested quercetin analogues.

In the PCA loading plot (Figure 3A), it can be observed that the majority of descriptors (9 out of 11) had a positive impact on PC1, while PSA and especially SOLY exerted a negative influence. On the other hand, MW and PSA had the highest positive influence on PC2, while the LOGP c-Hex and LOGP n-Oct descriptors had the dominant negative impact.

**Figure 4:**
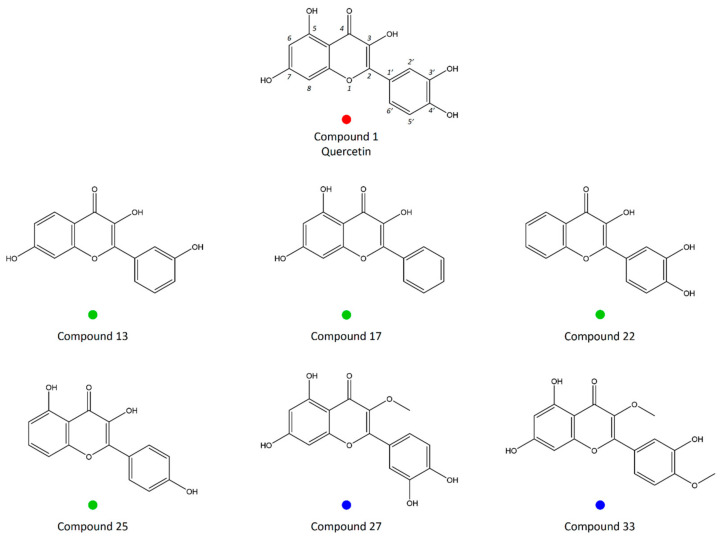
Chemical structures of quercetin and six analogues with the highest potential to cross the blood–brain barrier (compounds **13**, **17**, **22**, **25**, **27**, **33**).

Among 35 compounds (quercetin and 34 analogues), six compounds with the highest LgBB values, i.e., with the highest potential to permeate the BBB, were marked in the PCA score plot (Figure 3B), along with the quercetin itself. These quercetin analogues (compounds **13**, **17**, **22**, and **25**) are located in the lower-left segment of the PCA score plot, suggesting that intrinsic solubility and logP have a dominant influence on the ability of quercetin analogues to permeate the BBB. The exceptions are compounds **27** and **33**, which are are specific, since they are the only two quercetin analogues that are substrates for P-gp. Therefore, these compounds are marked differently in the PCA score plot. The chemical structures of quercetin and six quercetin analogues with the highest potential to permeate the BBB are demonstrated in Figure 4.

## 3. Discussion

Beneficial effects of quercetin in neurodegenerative disorders, such as Alzheimer’s disease (AD), Parkinson’s disease (PD), Huntington’s disease (HD), and cognitive impairment, have been reported in several in vitro and in vivo models. However, the clinical efficacy of quercetin in combating neurodegenerative diseases is limited due to its low oral bioavailability and particularly its low CNS distribution [4]. Numerous chemical and formulation strategies to surmount these obstacles have emerged [14], including the identification of quercetin analogues that possess similar pharmacological activity but more favorable pharmacokinetic characteristics using in silico techniques [15,16]. Similar methodology has already been applied in the development of other potentially novel drugs from herbal sources [17].

Computational methods can be used for many purposes in biomedical research, but when the predictions are used to make clinical recommendations, it is necessary to demonstrate the credibility of the model used, i.e., the key elements of verification, validation, and uncertainty quantification of mechanistic modeling and simulation should be defined [18]. In this study, various well-established and validated in silico methods were used in order to identify quercetin analogues that have higher BBB permeability and preserved binding affinity towards IPMK, which plays a significant role in several brain disorders. Quercetin analogues were tested for binding affinities against IPMK using a molecular docking technique, and it was demonstrated that 19 compounds were able to form thermodynamically more stable complexes with IPMK active sites when compared to quercetin, thus achieving stronger activity at the targeted enzyme. Geraldol, i.e., 3′-methoxy-3,7,4′-trihydroxyflavone (compound **30**) exerted the strongest interaction with this enzyme, and the hydrophobic interactions between the methoxy group in the structure of geraldol and valine and leucine residues were shown to contribute to the high binding affinity towards IPMK.

Geraldol is a 3′-O-methylated derivative of fisetin, i.e., 3,3′,4′,7-tetrahydroxyflavone (compound **8**), and it was identified as its active metabolite in mice, showing a higher pharmacological potency [19]. Fisetin was shown to enhance learning and memory, to decrease neuronal cell death, and to suppress the oxidative stress in brain, thus exerting several beneficial effects on neurodegenerative diseases, especially AD and PD [20]. These pharmacological effects of fisetin may be also the consequence of geraldol activity. In our study, both geraldol and fisetin had higher binding affinity towards IPMK in comparison to quercetin. It is already known that the methylation of flavonoids can change their physiochemical and pharmacological properties and that the methylation position may determine different functions. For example, sterubin (7-methoxy-5,3′,4′-trihydroxyflavanone) was found to be about fourfold more potent in the phenotypic screening assays than fisetin due to higher logP and membrane permeability when compared to fisetin [21]. It should be noted that neuroprotective effects of fisetin may be also explained by its binding with AD-associated amyloid-binding alcohol dehydrogenase, acetylcholinesterase, and β-site amyloid precursor protein-cleaving enzyme 1 (BACE1) enzymes, as previously predicted by molecular docking [22].

In order to predict the permeability of quercetin analogues through the BBB, several in silico parameters were taken into account. Molecular descriptors relevant to the membrane permeability in general (LOGP n-Oct and LOGP c-Hex), and BBB permeability in particular (LgBB) were calculated. According to the calculated values of these molecular descriptors, the vast majority of the examined compounds were found to be more lipophilic than quercetin. However, the calculated LgBB values were lower than −0.5 for all quercetin analogues, indicating insufficient brain permeation. Similar results were obtained using the SwissADME BOILED-Egg model as a simple discriminative technique to estimate passive BBB crossing. All quercetin analogues were outside the yolk region (Figure 2), where BBB-permeating compounds should be located.

Nevertheless, considering the wide range of calculated LgBB values (−3.311 to −1.263) and the widespread distribution of quercetin analogues in the BOILED-Egg plot, additional studies on the influence of their physicochemical characteristics, i.e., molecular descriptors, on BBB permeation, as well as further structural optimization, is necessary. Principal component analysis (PCA) is a well-known mathematical procedure used for data analysis and reduction, widely applied in various science fields. It is often used in the early stages of the drug discovery pipeline, for example, in quantitative structure–activity relationship (QSAR) studies, where it analyzes complex data matrices of molecular descriptors and transforms them into a simpler and smaller set of uncorrelated variables called principal components (PCs). As a result, we obtain a score plot that clusters the objects, in our case the compounds and loading plot that describes the contribution of molecular descriptors to the positioning of the objects in the score plot [23,24,25].

In the PCA score plot for 34 analyzed quercetin analogues (Figure 3B), compounds with the highest LgBB values (approximately −2 and higher), i.e., compounds with the highest BBB permeability potential, have been highlighted, and it can be observed that most of these compounds are clustered in the lower-left part of the score plot (compounds **13**, **17**, **22**, and **25**). All four compounds are trihydroxyflavones, containing two hydroxyl groups fewer than quercetin, and thus being more lipophilic. The loading plot in Figure 3A suggests the possible role of analyzed descriptors on the clustering of molecules. Specifically, descriptors related to intrinsic solubility (SOLY) and the ones related to lipophilicity (LOGP n-Oct and LOGP c-Hex) were mainly responsible for BBB permeability separation. However, the exceptions were compounds **27** and **33**.

Among these compounds with the highest BBB permeability, we can highlight compounds **17** and **22**, which already showed activity in several brain disorders. For example, in vitro studies showed that galangin (compound **17**) can efficiently inhibit human brain acetylcholinesterase, butyrylcholinesterase and 5-lipoxygenase [26]. It was also shown that galangin blocks thrombin-induced MMP-9 expression, thus being a candidate for the management of brain inflammatory diseases [27]. In vivo studies showed that 3′,4′-dihydroxyflavonol, i.e., 3,3′,4′-trihydroxyflavone (compound **22**) has a protective effect on brain ischemia–reperfusion injury in rats [28] and also reverses spatial learning and memory deficits resulting from transient global ischemia [29]. These studies confirm our PCA results, which clustered these compounds as possible candidates for treating neurological disorders, having better BBB permeability than quercetin. In addition, 3,7,3′-trihydroxyflavone (compound **13**), which is grouped with compounds that could have higher CNS distribution in comparison to quercetin, also has a higher affinity towards IPMK, which makes it a candidate for further investigation. A similar compound, 3′,4′,7-trihydroxyflavone, was shown to prevent hydrogen peroxide-induced apoptotic cell death by suppressing activation of the NF-κB pathway through oxidative stress in neuronal cells [30].

Quercetin–methyl conjugates have been demonstrated to have higher oral bioavailability in vivo than quercetin itself [31]. The methylated flavones were much more metabolically stable compared to non-methylated forms in pooled human liver S9 fraction, and they showed five- to eightfold higher apparent permeability through Caco-2 cells [32]. As previously stated, compound **27** (3-O-methylquercetin) and compound **33** (quercetin 3,4′-dimethyl ether) were not clustered with other quercetin analogues possessing higher LgBB values in the PCA score plot, but were grouped in the upper-right area of the plot. These compounds are specific since they are the only two analyzed quercetin analogues that are substrates for P-glycoprotein (P-gp). P-gp is a broad-spectrum efflux pump expressed in the apical membranes of hepatocytes and epithelial cells of the proximal tubule, and the luminal membrane of the small intestine and BBB. Compounds that interact with P-gp can be identified as substrates, modulators, and inhibitors. Substrates are susceptible to efflux by P-gp, thus having a reduced possibility of penetrating the BBB. P-gp reduces the potential cerebral concentrations of structurally unrelated compounds, including 50% of currently marketed drugs [33,34,35]. The methoxy group in the position C3, next to the C4 keto group, is considered to be responsible for these two compounds being P-gp substrates. This can lead to their low concentrations in CNS despite having the highest LgBB values, i.e., BBB permeability, among all tested quercetin analogues. In accordance with this, 3-O-methylquercetin was unable to exert neuroprotective effects in hydrogen peroxide-induced toxicity model using primary neuronal culture, in contrast to quercetin [36].

## 4. Methods and Materials

### 4.1. Protein and Ligand Preparation

The three-dimensional (3D) crystal structure of human IPMK in a complex with quercetin (PDB code: 6M89) [6], which was determined by X-ray diffraction at a resolution of 1.85 Å, was taken from the Protein Data Bank [37]. Ligand structures were retrieved from the ZINC database [38] by importing the structure of quercetin in SMILES format and using the structural similarity search method to obtain chemically related compounds. ZINC supports whole-molecule similarity search with SmallWorld using graph-edit-distance search, annotated with traditional ECFP4 and Daylight Tanimoto similarity values calculated [39]. The search parameters were set at 90% similarity, which resulted in the identification of 34 quercetin analogues in unionized form.

### 4.2. Molecular Docking Analysis

Docking analyses were performed using Molegro Virtual Docker (MVD) software version 6.0 [40]. Quercetin analogues in mol2 format and IPMK protein in pdb format were imported into the MVD program and solvent molecules were removed. The validation of the docking protocol was performed by “redocking” the co-crystalized quercetin in the structure of IPMK. The root mean square distance (RMSD) of the docked ligand was lower than 2 Å, confirming that docked quercetin was able to interact with the IPMK active site similarly to the preexisting co-crystallized quercetin when using this docking protocol.

Potential ligand binding sites for IPMK were predicted using MVD, and the cavity with a volume of 89.1 Å^3^ and a surface area of 294.4 Å^2^ containing the co-crystallized quercetin and encompassing all protein atoms within 10 Å of the co-crystallized ligand was used for further docking analyses. Different orientations of quercetin analogues were ranked based on their energy scores. Grid-based MolDock score (GRID) function with a grid resolution of 0.30 Å was used for all docking calculations and MolDock SE was used as a search algorithm. The number of runs was 10, while a population size of 50 and a maximum iteration of 1500 were used for parameter settings. Five poses for each ligand were generated, and the best pose was used for the ligand–protein interaction energy analysis.

### 4.3. In Silico Prediction of CNS Distribution

Molecular descriptors relevant to membrane permeability for quercetin and its analogues were predicted using VolSurf+ software version 1.0.4 (Molecular Discovery, Borehamwood, UK) [41]. LogP octanol–water (LOGP n-Oct) and logP cyclohexane–water (LOGP c-Hex) as the logarithms of the partition coefficient between n-octanol or cyclohexane and water were computed via a linear equation derived by fitting GRID-derived atom type to experimental data. The logarithm of the blood–brain barrier distribution (LgBB) was calculated and values lower than −0.5 indicated poor brain permeation.

Permeation through BBB was also predicted using the SwissADME web tool, which includes the “Brain Or IntestinaL EstimateD” permeation method (BOILED-Egg evaluation) [42]. BOILED-Egg is a graphical evaluation of human intestinal absorption as a function of the position of the small molecule in the WLOGP vs. TPSA plot. The white region of the BOILED-Egg depicts the high probability of passive absorption in the gastrointestinal tract, and the yellow region (yolk) describes the high probability of brain penetration (yolk and white areas are not mutually exclusive).

The interactions of quercetin and its analogues with P-glycoprotein (P-gp) were predicted using the PgpRules server [43]. PgpRules predicts substrate and inhibitory properties of compounds towards P-gp. The prediction is based on the classification and regression tree (CART) algorithm. The rules are calculated based on PubChem 2D fingerprints and RDKit descriptors.

Molecular descriptors indicating the intrinsic solubility (SOLY) and Caco-2 cell permeability (CACO2) were also calculated, and they are presented as supplementary material. Eventually, a high-throughput screening flag (HTSFlag) parameter was tested using VolSurf+ software 1.0.4 in order to assess the suitability of applied in silico methods for the investigated set of compounds, and this descriptor had a value of 0 for all tested quercetin analogues, indicating that they are suitable for in silico studies.

### 4.4. Principal Component Analysis (PCA)

The values of size/shape and physicochemical molecular descriptors for all data-set compounds were calculated using VolSurf+ software 1.0.4. Determined descriptors of the tested quercetin and its analogues were: molecular volume (V), molecular globularity (G), amphiphilic moments (A), critical packing parameter (CP), polarizability (POL), molecular weight (MW), logP octanol–water (LOGP n-Oct), logP cyclohexane–water (LOGP c-Hex), polar surface area (PSA), hydrophobic surface area (HSA), and intrinsic solubility (SOLY).

Principal component analysis (PCA) was performed in order to substitute the representation of the objects, from the initial representation in the form of the n original intercorrelated variables, into the new principal component (PC) coordinate space. PCs are quantified by their so-called loadings and scores, where scores are the new coordinates of the projected objects, and loadings reflect the direction concerning the original variables. Since the loading plot shows relations between variables, it was used to identify molecular descriptors that contribute to the positioning on the score plot. In the score plot, the grouping pattern of the tested compounds was analyzed and the potential outliers, i.e., compounds lying outside the Hotelling T^2^ ellipse, identified.

## 5. Conclusions

Using several in silico methods, it was shown that none of 34 analyzed quercetin analogues have sufficient BBB permeation. However, the higher concentrations of several identified compounds (**13**, **17**, **22** and **25**) are expected in the brain in comparison to quercetin itself. In addition, the application of the PCA statistical method in this study proved to be significantly beneficial in the analysis of data regarding the relationship between molecular descriptors and their properties. PCA scores and loading plots provided information about the influence of a single molecular descriptor on clustering the compounds regarding their structural characteristics. It was demonstrated that there needs to be a balance between the descriptors related to intrinsic solubility (SOLY) and the ones related to lipophilicity (LOGP n-Oct and LOGP c-Hex) in order to achieve the desired brain distribution.

In conclusion, by detecting the molecular descriptors with the strongest contribution to the BBB permeability of quercetin analogues, the synthesis of new compounds with desired properties reflected in favorable values of molecular descriptors is enabled. Therefore, this work can be considered a step toward the discovery and further development of quercetin analogues with enhanced brain distribution. However, further in vitro and in vivo studies are proposed to confirm these results and allow the use of quercetin analogues as neuroprotective agents in clinical settings.

## Figures and Tables

**Table 1:** Binding affinities towards inositol phosphate multikinase (IPMK) and molecular descriptors of lipophilicity and permeation across the blood–brain barrier for quercetin and its analogues.

No.	Compound	MolDock Score ^1^ [kcal/mol]	LOGP nOct ^2^	LOGP cHex ^2^	P-gp Substrate ^3^	P-gp Inhibitor ^3^	LgBB ^2^	BBB Permeant ^4^
1	ZINC03869685	−82.233	2.078	−5.609	No	No	−2.955	No
2	ZINC03874317	−82.088	1.815	−7.256	No	No	−3.224	No
3	ZINC05784821	−80.472	1.942	−5.924	No	No	−3.200	No
4	ZINC04098600	−80.627	2.255	−5.273	No	No	−2.865	No
5	ZINC06520226	−78.471	2.669	−4.283	No	No	−2.822	No
6	ZINC14436449	−81.632	1.783	−6.806	No	No	−3.311	No
7	ZINC06484604	−86.261	2.275	−4.212	No	No	−2.707	No
8	ZINC00039111	−86.966	2.518	−3.626	No	No	−2.552	No
9	ZINC06525297	−82.619	1.873	−5.635	No	No	−3.163	No
10	ZINC03869768	−81.960	2.238	−5.530	No	No	−2.745	No
11	ZINC00517261	−86.485	2.275	−4.212	No	No	−2.847	No
12	ZINC03875620	−80.734	2.570	−2.354	No	No	−2.615	No
13	ZINC00057845	−87.179	2.692	−3.660	No	No	−2.074	No
14	ZINC04731234	−76.973	2.600	−3.994	No	No	−2.837	No
15	ZINC01645590	−83.643	2.767	−0.957	No	No	−2.235	No
16	ZINC06018683	−88.909	2.472	−2.815	No	No	−2.522	No
17	ZINC00120273	−78.290	2.670	−2.890	No	No	−1.924	No
18	ZINC05998785	−75.133	2.275	−4.212	No	No	−2.822	No
19	ZINC06483609	−88.006	2.209	−4.462	No	No	−3.034	No
20	ZINC06483700	−90.616	2.337	−2.776	No	No	−2.667	No
21	ZINC06403375	−85.664	2.767	−0.957	No	No	−2.505	No
22	ZINC00039321	−74.655	2.936	−0.873	No	No	−1.773	No
23	ZINC03881558	−85.134	1.919	−6.491	No	No	−3.099	No
24	ZINC06411540	−85.910	2.730	−2.275	No	No	−2.333	No
25	ZINC00057752	−72.415	2.642	−2.664	No	No	−1.973	No
26	ZINC06536276	−82.025	2.033	−5.556	No	No	−2.964	No
27	ZINC05998596	−82.584	2.249	−5.092	Yes	No	−1.525	No
28	ZINC00008662	−82.625	2.518	−3.626	No	No	−2.400	No
29	ZINC02146994	−89.938	2.777	−0.793	No	No	−2.267	No
30	ZINC05732763	−91.827	2.715	−2.229	No	No	−2.513	No
31	ZINC48057104	−81.994	2.199	−4.587	No	No	−2.683	No
32	ZINC05733650	−75.462	2.464	−1.444	No	No	−2.376	No
33	ZINC05640267	−87.012	2.446	−3.695	Yes	No	−1.263	No
34	ZINC14644239	−84.261	2.003	−5.232	No	No	−2.699	No
35	ZINC06095498	−82.714	2.337	−3.738	No	No	−2.375	No

^1^ Molegro Virtual Docker; ^2^ VolSurf+; ^3^ PgpRules; ^4^ SwissADME.

## Data Availability

The data presented in this study are available in the article.

## Supplementary Materials

The following supporting information can be downloaded at https://www.mdpi.com/article/10.3390/ijms25010192/s1.
