# Supplementary material for: Principal Component Analysis (PCA) of Molecular Descriptors for Improving Permeation through the Blood–Brain Barrier of Quercetin Analogues
Source: Int J Mol Sci. 2023 Dec 22;25(1):192. doi: 10.3390/ijms25010192 (PMC10778702; doi:10.3390/ijms25010192)
Supplement: Supplementary file 1 [file ijms-25-00192-s001.zip › ijms-2761437-supplementary.pdf]

Table S1. VolSurf+ molecular descriptors on intrinsic solubility (SOLY) and Caco-2 cell permeability (CACO2).

No.	Compound	SOLY	CACO2
1	ZINC03869685	-2.399	-0.8823
2	ZINC03874317	-2.237	-1.1370
3	ZINC05784821	-2.293	-1.0994
4	ZINC04098600	-2.471	-0.8578
5	ZINC06520226	-2.790	-0.7962
6	ZINC14436449	-2.267	-1.0666
7	ZINC06484604	-2.782	-0.7663
8	ZINC00039111	-2.633	-0.6158
9	ZINC06525297	-2.327	-1.0619
10	ZINC03869768	-2.755	-0.6433
11	ZINC00517261	-3.012	-0.7081
12	ZINC03875620	-2.976	-0.5747
13	ZINC00057845	-2.626	-0.3732
14	ZINC04731234	-2.756	-0.7602
15	ZINC01645590	-3.280	-0.3501
16	ZINC06018683	-3.291	-0.4031
17	ZINC00120273	-2.691	-0.1319
18	ZINC05998785	-2.778	-0.6876
19	ZINC06483609	-3.227	-0.7331
20	ZINC06483700	-3.415	-0.4361
21	ZINC06403375	-3.534	-0.3873
22	ZINC00039321	-2.584	-0.0568
23	ZINC03881558	-2.661	-0.9236
24	ZINC06411540	-3.234	-0.3518
25	ZINC00057752	-2.712	-0.1557
26	ZINC06536276	-2.404	-0.8625
27	ZINC05998596	-3.743	-0.4022
28	ZINC00008662	-2.528	-0.5212
29	ZINC02146994	-3.677	-0.1951
30	ZINC05732763	-3.286	-0.4251
31	ZINC48057104	-2.273	-0.7936
32	ZINC05733650	-3.281	-0.4477
33	ZINC05640267	-3.988	-0.0879
34	ZINC14644239	-2.512	-0.6833
35	ZINC06095498	-2.656	-0.4165
